# Primary hepatic peripheral T-cell lymphoma: A case report

**DOI:** 10.3892/ol.2014.2119

**Published:** 2014-05-07

**Authors:** HUI-JUAN HU, MEI-YAN LIAO, YAN-JUAN QU

**Affiliations:** Department of Computed Tomography, Zhongnan Hospital of Wuhan University, Wuhan, Hubei 430071, P.R. China

**Keywords:** primary hepatic lymphoma, liver mass, computed tomography, magnetic resonance imaging

## Abstract

Primary hepatic peripheral T-cell lymphoma (PHL) is extremely rare. A case of primary hepatic peripheral T-cell lymphoma of a 59-year-old male is presented in the current study. PHL lesions are diagnosed by the existence of a hepatic mass, in the absence of lymphadenopathy, splenomegaly or bone marrow involvement associated with normal tumor markers. Treatment options are surgical resection and subsequent chemotherapy. Histopathological examination by immunohistochemical staining of the tissue biopsies at laparotomy confirmed a diagnosis of PHL.

## Introduction

The occurrence of primary hepatic lymphoma (PHL) is infrequent, and is responsible for <1% of all extranodal lymphomas (1,2). The pathological diagnosis is usually diffuse large B-cell lymphoma, and primary T-cell lymphoma of the liver is extremely rare with only a few cases reported in the literature (3), and is responsible for 5–10% of PHLs (4). In the present study, a case of primary hepatic peripheral T-cell lymphoma in a middle-aged male patient is reported with a brief review of the literature. Patient provided written informed consent.

## Case report

### Case presentation

A 59-year-old male patient presented to Zhongnan Hospital of Wuhan University (Wuhan, China), on May 17, 2013, with fatigue, weight loss of 20 kg and a three-day history of right upper abdominal pain. The patient had no history of fever, vomiting, night sweats, chest pain, icterus, diarrhea or stool blood loss. The general physical and chest examinations of the patient were unremarkable, except for right upper quadrant tenderness, with no peripheral lymphadenopathy. The past medical and personal histories of the patient were hypertension and hyperlipidaemia for 5 years, and diabetes mellitus for 2 years. Laboratory results included a hemoglobin level of 15.0 g/dl and a white blood cell count of 8.3×10^9^/l, with a normal differential. Further laboratory investigation revealed an alanine aminotransferase (ALT) level of 175 U/l and an aspartate aminotransferase (AST) level of 222 U/l, and other liver and renal function tests were within normal limits. Levels of serum α-fetoprotein (AFP), carcinoembryonic antigen (CEA) and other tumor markers were normal. Serology was negative for human immunodeficiency (HIV), syphilis antibody, hepatitis C (HCV) and hepatitis B (HBV) viruses. The patient had a serum lactate dehydrogenase (LDH) level of 441 UI/ml (normal range, 135–225 UI/ml), and the level of β2-microglobulin was normal (1.38 mg/l).

### Imaging

**Figure 1:**
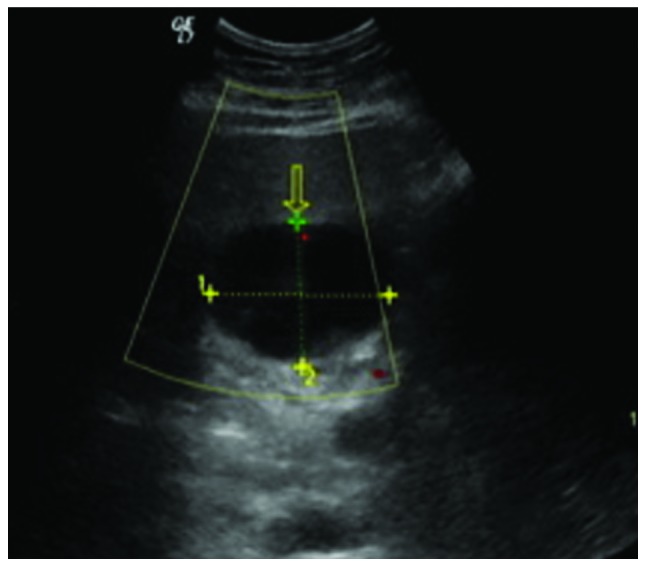
Abdomen Doppler ultrasound. A well-defined hypoechoic area (~53×39 mm) at the quadrate lobe of the liver, showing that the internal echo of the mass was heterogeneous.

**Figure 2:**
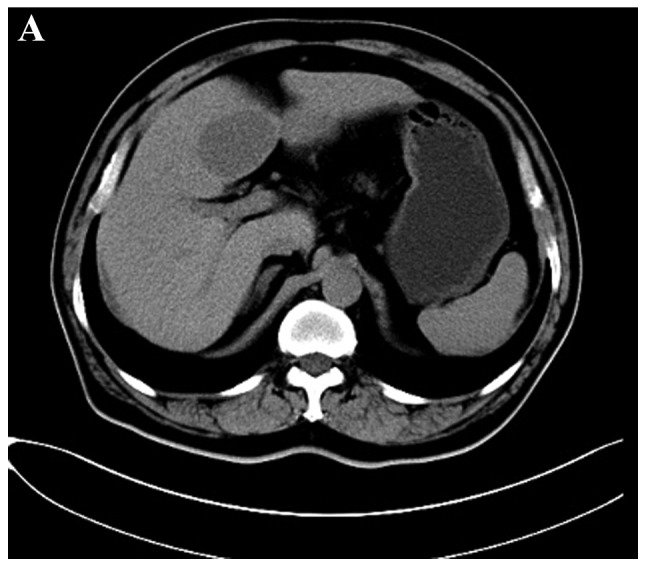

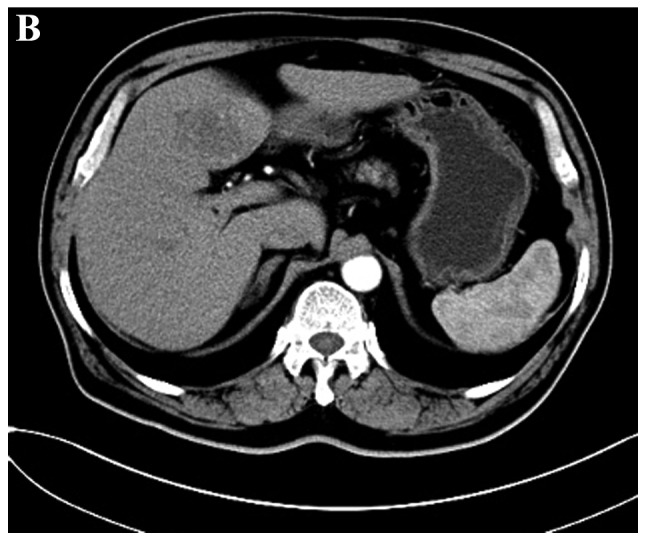

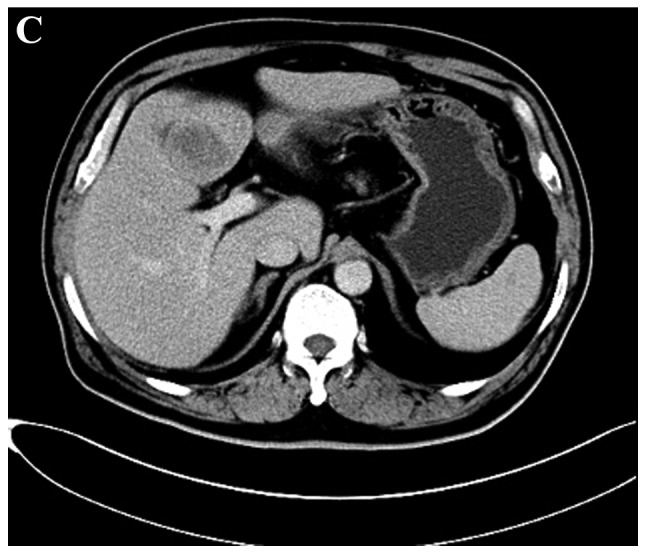

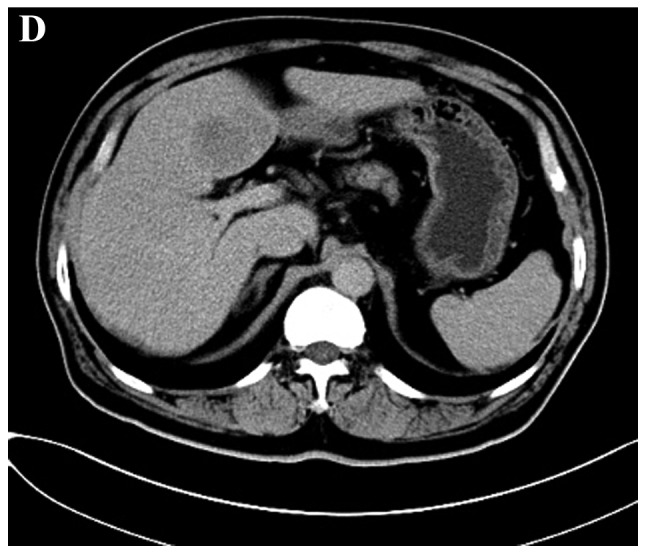
CT images of the lesion. (A) On the plain CT scan, an oval homogeneous hypodense mass with a distinct border is located in the quadrate lobe of the liver. (B) Arterial, (C) portal venous and (D) delayed phase CT scan showing slight and continued enhancement in the peripheral of the mass. CT, computed tomography.

**Figure 3:**
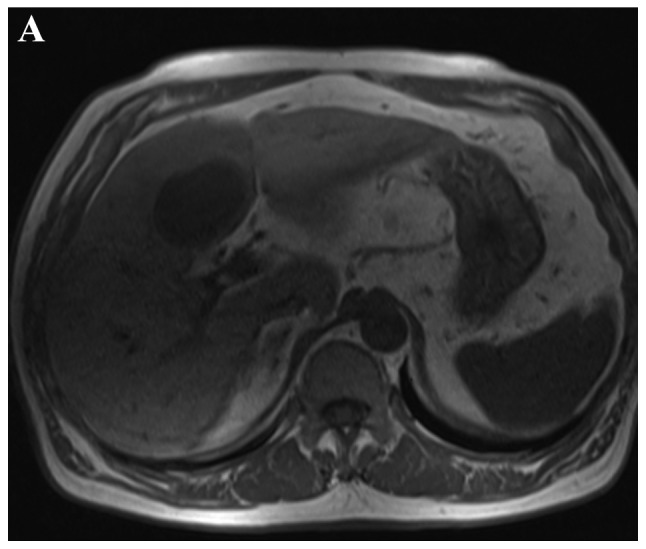

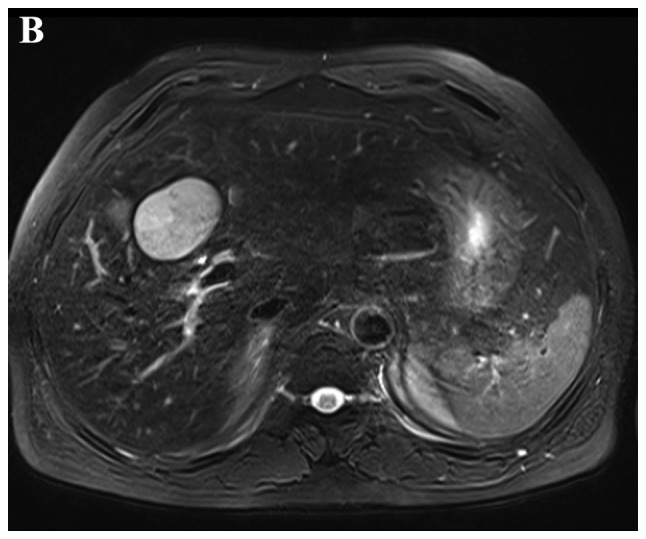

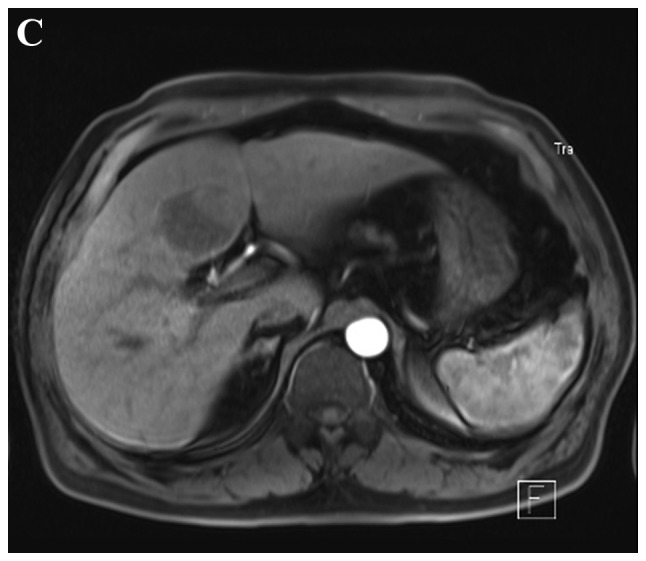

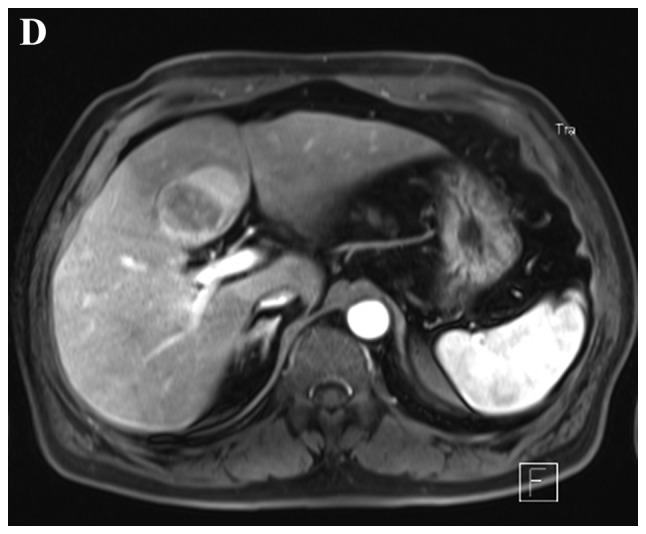

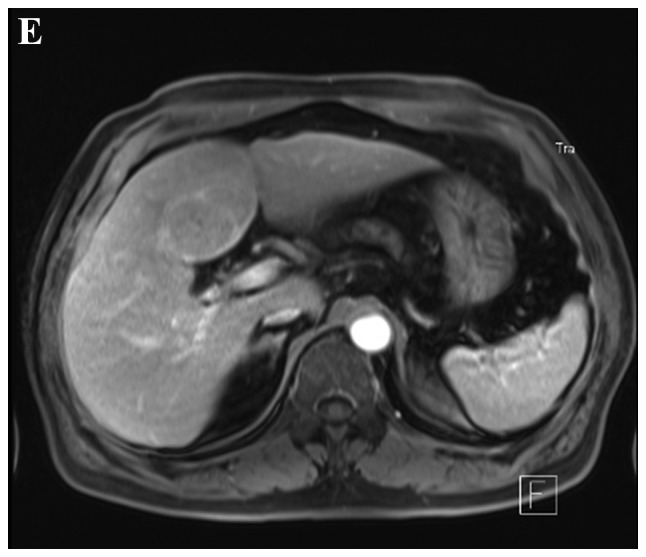
MRI images of the lesion. The solitary lesion at the fourth hepatic segment is (A) hypointense on T1WI and (B) hyperintense on T2WI. The dynamic gadolinium-diethylenetriaminepentaacetic acid MRI protocol showing a (C) mild ring-like enhancement during arterial phase, (D) continued and prominent enhancement in portal venous phase, and (E) that the enhancement of the tumor has decreased in the delayed phase, and that the septum is enhanced. MRI, magnetic resonance imaging; WI, weighted image.

The chest X-ray did not show any abnormality. Abdominal ultrasonography (US) showed a well-defined hypoechoic mass of 53×39 mm in the quadrate lobe of the liver, and the internal echo was heterogeneous (Fig. 1). Diagnostic imaging was performed by computed tomography (CT) and a magnetic resonance imaging (MRI) scan of the abdomen. On abdominal CT scan (Siemens Somatom Definition; Siemens Medical Solutions, Erlangen, Germany), an oval homogenous and low-density mass that measured ~40×58 mm in the largest section with a distinct border located in the quadrate lobe of liver was demonstrated prior to contrast material injection (Fig. 2A). On triple-phase (arterial, portal venous and delayed phase) iodinated contrast-enhanced CT scan, a slight and persistent ring-like enhancement was visible in the peripheral but not in the entire tumor, the center of which was minimally enhanced (Fig. 2B–D). Supplemental abdominal MRI with contrast medium (Siemens Trio 3.0T; Siemens Medical Solutions) showed a homogeneous and distinct solitary lesion at the fourth hepatic segment, which had a low signal intensity on T1 weighted image (WI) and a high signal intensity on T2WI (Fig. 3A and B). The dynamic gadolinium-diethylenetriaminepentaacetic acid MRI protocol showed a mild ring-like enhancement during the arterial phase, which continued and showed a prominent enhancement in the portal venous phase. The enhancement of the tumor decreased in the delayed phase and showed the enhancement of the septum (Fig. 3C–E).

### Surgery and pathological analysis

**Figure 4:**
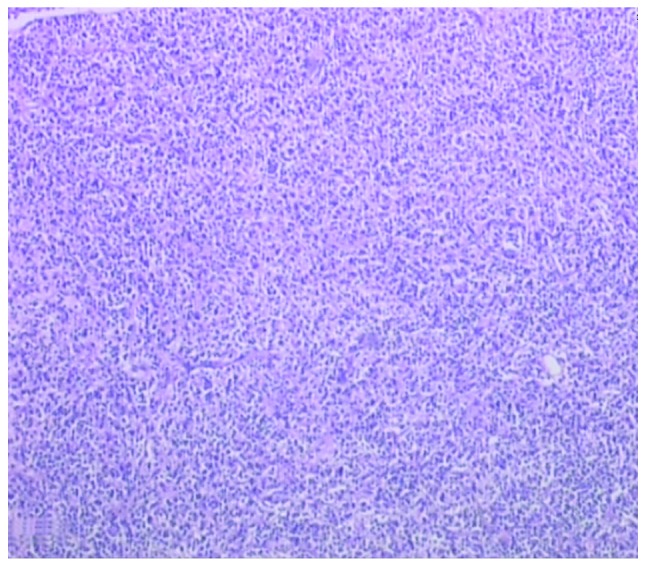
Photomicrograph of the operated specimen showing a diffuse infiltrate with medium-to-large-sized lymphoid cells indicating lymphoma (hematoxylin and eosin stain; magnification, ×100).

**Figure 5:**
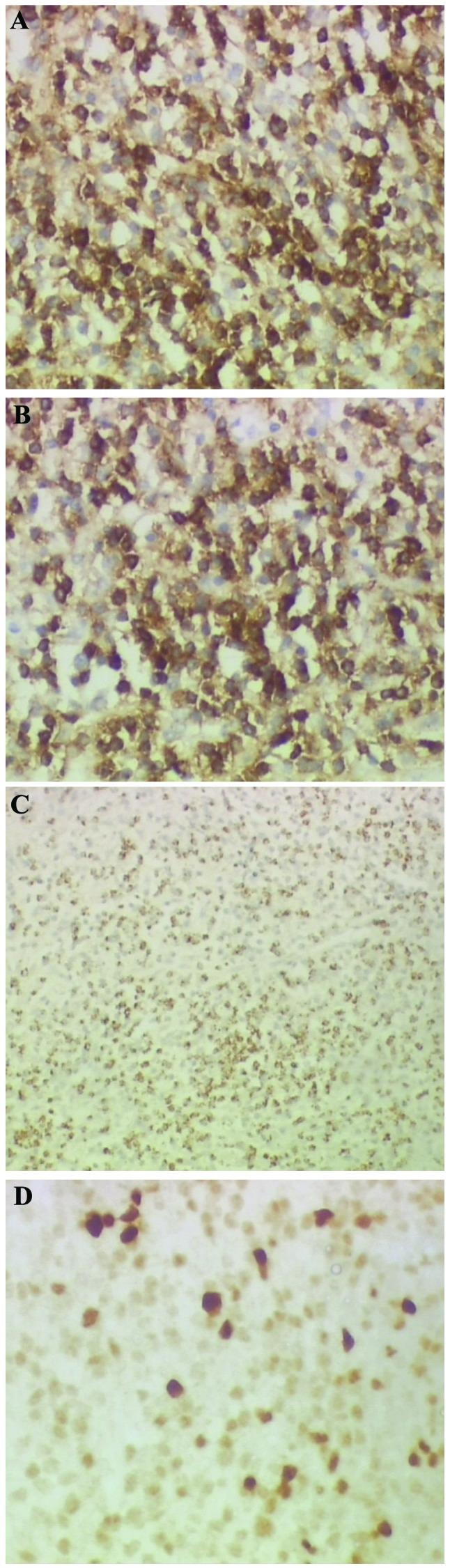
Immunohistochemistry of the specimen showing that the cells have stained positive for (A) CD3, (B) CD5, (C) TIA-1 and (D) multiple myeloma oncogene 1. Magnification, ×400. CD, cluster of differentiation.

The patient underwent total resection of the mass. Preoperatively, the mass measured 60×40 mm and it was lobulated, well-defined and had necrosis at the centre. Histopathological analysis of the tissue disclosed a diffuse infiltrate with medium-to-large-sized lymphoid cells indicative of lymphoma (Fig. 4). Immunohistochemistry of the tumor cells showed reactivity for cluster of differentiation 3 (CD3) (Fig. 5A), CD5 (Fig. 5B), TIA-1 (Fig. 5C) and multiple myeloma oncogene 1 (Fig. 5D), and was negative for CD20, CD79, activin receptor-like kinase-1, CD30, CD10, myeloperoxidase, B-cell lymphoma 6 and smooth muscle actin. The Ki-67 index of those lymphoid cells was 30%.

### Chemotherapy and follow-up

Following a discussion of the risks of chemotherapy and radiotherapy with the patient and his family, the patient received chemotherapy (CHOP: 1500 mg cyclophosphamide, 150 mg epirubicin-adriamycin, 2 mg vincristine and 100 mg prednisone). The courses of chemotherapy were administered every 21 days. Subsequent to receiving six cycles of chemotherapy, the patient underwent radiotherapy of liver (Dt = 30Gy/15F). During the treatment period with chemotherapy and radiotherapy, there were no major complications. The patient has undergone follow-up for almost 1 year with no evidence for recurrence of the disease.

## Discussion

According to the criteria by Caccamo *et al*, PHL is established as being a lymphoma with only the involvement of the liver at presentation. Six months after the diagnosis, other tissues can be involved, including the spleen, lymph nodes, peripheral blood, bone marrow or other tissues (5). PHL is notably rare, it constitutes 0.4% of cases of extranodal non-Hodgkin’s lymphoma (NHL), and only ~0.016% of all cases of NHL (6). The most common histological type of PHL is diffuse large B-cell lymphoma, and primary hepatic T-cell lymphoma is extremely rare with only a few cases reported in the literature, which are responsible for 5–10% of PHLs (3).

The etiology of PHL is unknown, despite certain possible etiological factors having been proposed, including HCV (7–9), HBV (10) and Epstein-Barr virus (EBV) (11). HCV infection has been identified in 20–60% of PHL patients. The persistent correlation with HCV indicates that this virus may play a role in PHL pathogenesis (7,12). PHL has been noted to occur in patients with immune suppression, such as HIV or human T-lymphotropic virus infections, systemic erythematous lupus and immunosuppressive therapy (2,11). However, the patient of the present study had neither HCV infection nor signs of immunodeficiency, due to negative serology for HIV, HBV, HCV and EBV active infection. Therefore, it is speculated that PHL could also occur in patients without any liver disease.

PHL commonly occurs at 50–60 years of age, with a male/female ratio of 2–3/1 (13). PHL has non-specific clinical manifestations. The most frequent symptom at presentation is abdominal pain or discomfort, occurring in 39–70% of patients (3), and other symptoms include fever, loss of weight and night sweats (also known as ‘B’ symptoms), nausea, vomiting, asthenia or itching. The main laboratory findings are abnormal hepatic functional enzymes, including AST, ALT, bilirubin, γ-glutamyl transferase, ALP, and LDH. Liver function tests are abnormal in <70% of cases and LDH is elevated in 30–80% of patients (2,11). Another study has also revealed that the dynamic change of serum LDH could serve as a diagnostic marker (14), but its use is limited due to poor specificity. β2-microglobulin, a well-described prognostic marker in lymphoma, is elevated in >90% of patients (12). AFP and CEA are tumor markers that are present at normal levels in ~100% of patients with PHL, which assists the differential diagnosis (12,15). In the present case, the levels of serum LDH, ALT and AST were elevated, those of AFP and CEA were normal, and the level of β2-microglobulin was normal.

At presentation, PHL may be a solitary lesion, multiple lesions or it may diffuse infiltration of the liver (16). The most common manifestation is a solitary lesion, and the diffuse infiltration is rare and indicates a worse prognosis. The imaging appearance of hepatic lymphoma is non-specific and, on ultrasound, the lesions usually appear hypoechoic with no typical vascularization pattern (3,17). PHL lesions appear as hypoattenuating in CT scans, which may have a low-intensity central area with no enhancement following the administration of an intravenous contrast in half the cases, patchy enhancement in 33% of patients and a ring of enhancement in ~25% of cases (3,17,18). Classically, MRI findings in PHL are described as ‘hypointense’ or ‘isointense’ on T1WI, and ‘hyperintense’ on T2WI (3,19). The imaging findings of hepatic lymphoma in the present case were similar to previous studies. Pre-contrast CT and MRI scans revealed that the mass was homogeneous and had a well-defined margin. Contrast-enhanced CT and MRI showed a ring-like enhancement.

Due to the rarity of this disease, non-specific clinical symptoms and laboratory and radiological manifestations, the diagnosis of PHL is extremely difficult. PHL may be confused with other diseases, including primary hepatic carcinoma, metastases and focal nodular hyperplasia. Laboratory and imaging findings are extremely helpful in differentiating between PHL and these diseases. Primary hepatic carcinoma appears as hyperechoic lesion in ultrasound, and CT scans show prominent arterial enhancement and iso- or hypodense on portal venous and delayed phases. The level of AFP is often elevated and the hepatic metastases have the history of a primary tumor generally. Focal nodular hyperplasia (FNH) usually appears hypo- or isodense on CT, and isointense on MRI. FNH is fairly homogeneous except for the central scar, which typically is hypodense on CT and T2-bright on MRI. The central scar is extremely specific. FNH shows rapid uptake of contrast in the arterial phase with a rapid return to near-normal enhancement in the portal venous and delayed phases. The central scar may enhance slightly in the delayed phase (20). However, PHL presents hypoechoic in US and low density in the CT scan. PHL shows no enhancement, minimally patchy or ring-like enhancement in contrast-enhanced CT, and delayed enhancement in the portal venous and delayed phases. The level of AFP and other tumor markers are normal. As the patient of the present study showed, the lesion was hypoechoic on ultrasound and low-density, minimally ring-enhancing on CT scan. For the MRI scan, the lesion presented a low signal intensity on T1WI and high signal intensity on T2WI. Combining the clinical and laboratory features, the diagnosis of PHL can be speculated. However, the definite diagnosis requires histological results by liver biopsy or surgical resection and the absence of lymphoproliferative disease outside the liver. The patient in the present case underwent surgical resection, and liver biopsy stained with specific immunohistochemical stains confirmed the diagnosis of PHL.

The optimal treatment of PHL has not yet been defined, however surgical treatment, radiotherapy and chemotherapy have been reported as treatment modalities both alone and in combination (21). It has been reported that surgical resection alone or in combination with chemotherapy may be a good treatment option for low-volume localized PHL (3,22). The patient of the present study employed surgical treatment and subsequent chemotherapy and radiotherapy in combination.

The majority of patients with PHL present with a poor prognosis. The median survival time for all patients is 15.3 months; however, the variation is wide and the reported survival time ranges from 3 to 123.6 months (11). In specific reports, the prognosis has been linked to the pattern of liver involvement (23) and the pathological subtype (3), and it is known that patients with unfavorable histologies have a low survival rate. The study by Emile *et al* (23) observed that in patients with nodular hepatic involvement, 1- and 3-year survival rates were 70 and 57%, respectively; however, when the liver was diffusely involved, the 1- and 3-year survival rates dropped to 38 and 18%, respectively. Therefore, it can be deduced that the patients with nodular involvement of the liver will have a longer survival rate. Yang *et al* (24) revealed that postoperative chemotherapy was the only significant prognostic factor that influenced survival rate. Noronha *et al* (3) reported that a patient, who was alive 5 years following the initial diagnosis, was treated with surgery followed by chemotherapy and radiation. A study by Lei (2) indicated that adjuvant chemotherapy subsequent to surgery should be considered for treatment of patients with localized disease to prevent recurrence. Therefore, we believe that a good prognosis can be achieved by early surgery combined with chemotherapy in patients with localized disease (such as solitary nodular PHL) and favorable histology

In conclusion, PHL is a notably infrequent disease, which lacks established imaging, clinical and biochemical markers. The diagnosis is difficult, as it is impossible to differentiate a single non-Hodgkin hepatic lesion from a metastatic nodule only by imaging techniques, particularly in the case of a history of tumor in a patient with unremarkable physical examination and no B symptoms. Biopsy or surgical resection should be performed when possible in case of an isolated hepatic nodule with radiological malignant aspects, particularly when serum tumor markers or biochemistry are not informative, as only histology can ensure an accurate differential diagnosis.
